# Complete Genome Sequence of Papaya Ringspot Virus Isolated from Genetically Modified Papaya in Hainan Island, China

**DOI:** 10.1128/genomeA.01056-15

**Published:** 2015-09-10

**Authors:** Guangyuan Zhao, Pu Yan, Wentao Shen, Decai Tuo, Xiaoying Li, Peng Zhou

**Affiliations:** Key Laboratory of Biology and Genetic Resources of Tropical Crops, Ministry of Agriculture, Institute of Tropical Bioscience and Biotechnology, Chinese Academy of Tropical Agricultural Sciences, Haikou, Hainan, China

## Abstract

The complete genome sequence (10,326 nucleotides) of a papaya ringspot virus isolate infecting genetically modified papaya in Hainan Island of China was determined through reverse transcription (RT)-PCR. The virus shares 92% nucleotide sequence identity with the isolate that is unable to infect PRSV-resistant transgenic papaya.

## GENOME ANNOUNCEMENT

Papaya ringspot virus (PRSV), a filamentous flexuous rod virus (760 to 800 × 12 nm) with a single-stranded positive-sense RNA as its genome (1, 2) belongs to the genus *Potyvirus* in the family *Potyviridae* (3). PRSV is naturally transmitted via aphids in a nonpersistent manner (4). It is also transmissible by mechanical inoculation (3). PRSV infects mainly papaya and cucurbits in the ﬁeld. It is the cause of a destructive disease and a major limiting factor for papaya and cucurbit cultivation worldwide (3, 5, 6).

Up-to-date genetic engineering is the most successful approach to control PRSV (3, 7). The PRSV-resistant genetically modified (GM) papaya has been commercially grown in Hawaii since 1998 and has played the major role in saving the papaya industry from economical demise (3, 5, 7–9). China approved one new PRSV-resistant GM papaya, Huanong No. 1, for commercialization in 2006 (10). Huanong No. 1 was transformed with the replicase gene of the PRSV strain from southern China. No breakdown of resistance occurred in the replicase-silenced GM papaya plants in the first 5 to 6 years (11). However, Huanong No. 1 showed less resistance to PRSV in recent years. In this study, the complete genomic sequence of the PRSV isolate infecting Huanong No. 1 GM papaya in Hainan Island (southern China) was obtained.

Leaves showing distorted and mosaic symptoms from Huanong No. 1 GM papaya were collected for total RNA isolation using the TRIzol reagent (Invitrogen, USA). The first-strand cDNA was synthesized using the TaKaRa RNA PCR kit (AMV) version 3.0 kit (Dalian, China) with oligo-dT as primers. Four primer pairs were designed to produce overlapping amplicons spanning the PRSV genome sequence according to the formerly cloned complete sequences of PRSV HN1 isolate (HQ424465). These four overlapping DNA fragments, ranging in size from 2,015 to 3,177 nucleotides (nt), were PCR amplified and cloned into pMD 18-T vector (TaKaRa, Dalian, China). The independent clones of each fragment were picked up to be sequenced by Invitrogen (Shanghai, China). The complete genome sequence (10,326 nt) was assembled using the four overlapping sequences, and this isolate infecting GM papaya in Hainan, China, was named PRSV-HN2. A BLAST search using the full genome sequence indicated that the PRSV-HN2 isolate showed 81% to 92% nucleotide sequence identities to known PRSV sequences. The isolate had the highest homology (92%) to the other three Hainan PRSV isolates (EF183499 [12], HQ424465 and KF734962 [13]) and had the lowest homology (81%) to the Hawaii isolate EU126128. The Hainan PRSV isolate EF183499, previously cloned in our lab (12), was unable to infect PRSV-resistant transgenic papaya. The complete genome sequence of PRSV-HN2 will facilitate research on the mechanism of the breakdown of PRSV resistance in GM papaya (14) and on the effect of GM papaya on PRSV evolution.

### Nucleotide sequence accession number. 

The full genomic sequence of PRSV Hainan isolate infecting GM papaya was deposited in GenBank under the accession number KF791028.
